# Deconvolving multiplexed protease signatures with substrate reduction and activity clustering

**DOI:** 10.1371/journal.pcbi.1006909

**Published:** 2019-09-03

**Authors:** Qinwei Zhuang, Brandon Alexander Holt, Gabriel A. Kwong, Peng Qiu

**Affiliations:** 1 School of Biological Sciences, Georgia Institute of Technology, Atlanta, Georgia, United States of America; 2 Wallace H. Coulter Department of Biomedical Engineering, Georgia Tech College of Engineering and Emory School of Medicine, Atlanta, Georgia, United States of America; 3 Parker H. Petit Institute of Bioengineering and Bioscience, Georgia Institute of Technology, Atlanta, Georgia, United States of America; 4 Institute for Electronics and Nanotechnology, Georgia Institute of Technology, Atlanta, Georgia, United States of America; 5 Integrated Cancer Research Center, Georgia Institute of Technology, Atlanta, Georgia, United States of America; 6 Georgia ImmunoEngineering Consortium, Georgia Tech and Emory University, Atlanta, Georgia, United States of America; University of Richmond, UNITED STATES

## Abstract

Proteases are multifunctional, promiscuous enzymes that degrade proteins as well as peptides and drive important processes in health and disease. Current technology has enabled the construction of libraries of peptide substrates that detect protease activity, which provides valuable biological information. An ideal library would be orthogonal, such that each protease only hydrolyzes one unique substrate, however this is impractical due to off-target promiscuity (i.e., one protease targets multiple different substrates). Therefore, when a library of probes is exposed to a cocktail of proteases, each protease activates multiple probes, producing a convoluted signature. Computational methods for parsing these signatures to estimate individual protease activities primarily use an extensive collection of all possible protease-substrate combinations, which require impractical amounts of training data when expanding to search for more candidate substrates. Here we provide a computational method for estimating protease activities efficiently by reducing the number of substrates and clustering proteases with similar cleavage activities into families. We envision that this method will be used to extract meaningful diagnostic information from biological samples.

## Author summary

The activity of enzymatic proteins, which are called proteases, drives numerous important processes in health and disease: including cancer, immunity, and infectious disease. Many labs have developed useful diagnostics by designing sensors that measure the activity of these proteases. However, if we want to detect multiple proteases at the same time, it becomes impractical to design sensors that only detect one protease. This is due to a phenomenon called protease promiscuity, which means that proteases will activate multiple different sensors. Computational methods have been created to solve this problem, but the challenge is that these often require large amounts of training data. Further, completely different proteases may be detected by the same subset of sensors. In this work, we design a computational method to overcome this problem by clustering similar proteases into "subfamilies", which increases estimation accuracy. Further, our method tests multiple combinations of sensors to maintain accuracy while minimizing the number of sensors used. Together, we envision that this work will increase the amount of useful information we can extract from biological samples, which may lead to better clinical diagnostics.

## Introduction

Proteases are multifunctional enzymes that hydrolyze peptide bonds and are responsible for maintaining health in processes ranging from immunity to blood homeostasis, but are also drivers of diseases, including cancer and sepsis [1–10]. The ability to quantify the activity of proteases–of which there are >550 –in humans on a larger scale may provide valuable biological information, leading to improved diagnostic and therapeutic technologies. While Next Generation Sequencing technologies provide the ability to rapidly assess mRNA transcript levels of proteases, previous studies have shown a lack of correlation between expression and enzyme activity [11–13]. For this reason, countless platforms have been developed to sense and modulate protease activity both *in vivo* and *in vitro*, with the potential to extract useful physiological information [10, 14–29]. However, to completely resolve an individual's protease landscape (i.e., >550 proteases) with current technology would require a library of equal size, assuming all substrates are orthogonal (i.e., each protease hydrolyzes a unique substrate), which is impractical at this scale (i.e., on the order of 10^2^). Further, current activity probes require experimental knowledge of protease-substrate specificity [30], which is difficult to completely map out because proteases are promiscuous [8], which means one protease is capable of hydrolyzing multiple different substrate sequences. Therefore, independent protease signatures become convolved when attempting to detect multiple proteases simultaneously, making it difficult to quantify the relative activity of each protease [31]. Previous studies have successfully developed computational algorithms to parse these signatures [32], but these methods may become complicated when applied to proteases with similar signatures in terms of their activities against substrates.

Here, to create a means for deconvolving protease signatures we develop a method, which requires limited prior knowledge of protease-substrate specificity. We demonstrate this method on a subset of blood proteases, including complement (e.g., C1r, MASP2, Factor D, etc.) and coagulation (e.g., Factor IIa, XIa, etc.) proteases, which display a high-degree of promiscuity and are involved in a range of hematological and immune disorders (i.e., clotting disorders, complement deficiencies, etc.) [33, 34]. To overcome the challenge of scaling to larger numbers of proteases, we use this method to improve experimental design by reducing the size of the substrate library. Furthermore, we cluster proteases with similar substrate activities into families, while maintaining high estimation accuracy. Under this framework, we lay the groundwork for understanding multiplexed protease-substrate signatures on a large scale, which may enable the future use of Massively Multiplexed Activity (MMA) libraries.

## Methods

To improve experimental design for deconvolving protease composition of protease mixtures, we developed pipelines for estimating kinetic parameters from real experimental data, and simulating *in silico* experimental data. In this Method section, we introduce the individual components of the pipeline (Method "Cleavage dynamics approximation model" to "Quantifying estimation accuracy via Root-Mean-Square Error (RMSE)"). We then apply the pipeline to optimize the selection of substrates and cluster proteases into families (Method "Evaluating deconvolution performance and optimizing substrate selection").

The overall strategy for the deconvolution analysis consists of two optimization steps. The first step consists of learning the cleavage dynamics of every combination of one protease and one substrate by optimizing kinetic parameters for a modified Michaelis-Menten model [35, 36] (see details in Method "Modified Michaelis-Menten kinetics with saturation" and "Estimating kinetic parameters in the single-protease-single-substrate setting"). We then apply the kinetic parameters learned in the first step to estimate the mixing coefficients, which represent the individual concentrations of proteases in a mixture (see details in Method "Estimating mixing coefficients with multiple proteases "). In the case where a sufficient number of substrates are measured to deconvolve all individual proteases in a mixture, we screen for the optimal subset of substrates in order to reduce the required number of substrates. When highly correlated proteases exist in the mixture, which would require an impractically large number of substrates for deconvolution, we cluster the proteases into families via hierarchical clustering to enable deconvolution based on a reasonable number of substrates and achieve a higher accuracy at a lower resolution (Method "Evaluating deconvolution performance and optimizing substrate selection").

### Recombinant protease activity assay

We tested a total of seven recombinant proteases in the activity assays. Complement proteases C1r (purity >90%), C1s (purity >95%), Complement Factor D (purity >90%), and Complement Factor I (purity >90%) were purchased from Sigma Aldrich. Complement protease MASP2 (purity >97%) was purchased from Biomatik. Coagulation proteases Factor IIa (purity >95%) and Factor XIa (purity >95%) were purchased from Haematologic Technologies. Initially, twenty peptide substrate sequences were curated from the literature, which represented discoveries from phage display screens as well as sequencing of physiological substrates [37–40]. We performed an initial screen with complement proteases which cleave after an arginine residue to identify the seven sequences used in these experiments (**Fig A in S1 Text**). To obtain the recombinant protease activity data, we first conjugated seven different c-terminus cysteine synthetic peptide substrates to amine functionalized 2 μm magnetic microparticles with SIA (i.e., succinimidyl iodoacetate), an amine-thiol crosslinker. The n-terminus of the peptides each contains one of seven unique glu-fib mass barcodes (**Table 1**). We then incubated a cocktail of these seven substrates (> 50 nM) with each of the seven recombinant proteases individually at 37°C on a spinner in PBS. At various time points between 0 and 400 minutes, we used a magnetic separator to remove the microparticles from the supernatant, which contained the hydrolyzed substrates plus mass barcodes. To provide a unique mass encoding for each of the seven substrates, we produced a family of mass reporters from Glu-fib with an isobaric mass-encoding strategy [41, 42]. By this method, all mass tags share the same parent mass so that peptides can be efficiently collected (i.e., MS-1) during tandem mass spectrometry (MS/MS), but can be differentiated after ion fragmentation (i.e., MS-2). Due to the fact that Glu-fib fragments into C-terminal y-type ions, we made mass codes centered on the y6 ion (i.e., GFFSAR). For each of the mass barcodes, we enriched the GFFSAR region with heavy amino acids, which resulted in sequences that varied by 1 Da each. To cancel out the resulting mass shifts, we balanced the remaining region (i.e., EGVNDNEE) by isotope enrichment. Mass spectrometry was performed by Syneos Health (Morrisville, NC) to quantify the amount of cleaved substrate at each time point. To summarize this method, 100 μL of sample volume and 25 μL of internal standard solution were UV-treated for 2 hours, using a UVP Analytik Jena UV Crosslinker CL-1000 oven. Sample cleanup was achieved using Mixed-mode anion exchange solid phase extraction. Chromatographic separation was achieved using a Waters XBridge C18 column, with the mobile phase composed of 0.1% formic acid in water and acetonitrile/trifluoroethanol. A gradient of 5% to 60% organic content at 0.6 mL/min over 3 minutes was employed. Analytes were analyzed using an AB Sciex 6500+ triple quadrupole mass spectrometer monitoring in MRM mode with an electrospray source set to positive ion mode. The total instrument run time was 5 minutes.

**Table 1:** Mass-barcoded peptide substrate sequences. Table describing the sequences of the mass-barcoded peptide substrates along with their chemical modifications. ANP was used as a photocleavable linker to enable rapid detachment from the microparticles. 5-FAM was used for rapid quantification via fluorescence. Isotope enrichment modifications were used to distinguish mass barcodes for quantification with mass spectrometry.

Substrate Name	Peptide sequence (N terminus on left)*	Modifications**
CC01	e(*aa)(*aa)ndneeGFFsAr(ANP)K(5-FAM)GGLQRIYKC	1st *aa = Gly(13C2); 2nd *aa = Val(U13C5,15N)
CC02	eG(*aa)ndneeGF(*aa)s(*aa)r(ANP)K(5-FAM)GGKSVARTLLVKC	1st *aa = Val(U13C5,15N); 2nd *aa = Phe(15N); 3rd *aa = Ala(15N)
CC03	e(*aa)(*aa)ndneeGFFs(*aa)r(ANP)K(5-FAM)GGQRQRIIGGC	1st *aa = Gly(U13C2,15N); 2nd *aa = Val(15N); 3rd *aa = Ala (U13C3,15N)
CC04	e(*aa)Vndnee(*aa)FFs(*aa)r(ANP)K(5-FAM)GGKYLGRSYKVC	1st *aa = Gly(13C2); 2nd *aa = Gly(13C2); 3rd *aa = Ala(U13C3,15N)
CC05	eGVndnee(*aa)(*aa)Fs(*aa)r(ANP)K(5-FAM)GGGLQRALEIC	1st *aa = Gly(U13C2,15N); 2nd *aa = Phe(15N); 3rd *aa = Ala(U13C3,15N)
CC06	e(*aa)(*aa)ndnee(*aa)(*aa)(*aa)s(*aa)r(ANP)K(5-FAM)GGKTTGGRIYGGC	1st *aa = Gly(13C2); 2nd *aa = Val(U13C5,15N); 3rd *aa = Gly(U13C2,15N); 4th *aa = Phe(15N); 5th *aa = Phe(15N); 6th *aa = Ala(15N); still include ANP and K5-FAM
CC07	eG(*aa)ndnee(*aa)(*aa)Fs(*aa)r(ANP)K(5-FAM)GGQARGGSC	1st *aa = Val(U13C5,15N); 2^nd^ *aa = Gly(U13C2,15N); 3rd *aa = Phe(15N); 4th *aa = Ala(U13C3,15N)

*ANP = Photocleavable linker 3-Amino-3-(2-nitrophenyl)propionic acid

*5-FAM = 5—Carboxyfluorescein

**Modifications represent heavy amino acids (i.e., isotope enrichment)

### Cleavage dynamics approximation model

Michaelis-Menten kinetics as the approximation of cleavage dynamics

$$\frac{d[S]}{dt}=-[\alpha ]V\frac{{\left[S\right]}^{n}}{{\left[S\right]}^{n}+K^{n}}$$ (1)

                              [*S*]: the remaining amount uncleaved substrate, [*S*] ∈ [0,1], [*S*]_*t = 0*_ = 1

                              *V*: the maximal rate of the reaction at the saturating substrate concentration

                              *K*: the substrate concentration when the reaction rate reaches half of *V*

                              *n*: the order of reaction

                              [*α*]: concentration of a protease

We use the Michaelis-Menten kinetics as the base model to approximate the cleavage dynamics [35, 36]. We add the mixing coefficient [*α*] (i.e., the concentration of a protease) is added to the original Michaelis-Menten model. On the left-hand side (LHS) of Eq (1), *d[S]/dt* represents the rate of change of the remaining uncleaved substrate. At t = 0, *d[S]/dt* is negative, which means that the substrate is being cleaved and [*S*] will decrease. As [*S*] decreases, the cleaving process slows down until *[S]* arrives at 0, where the reaction stops due to the depletion of the uncleaved substrates. However, in real experimental data, we noticed persistent non-zero saturation levels of uncleaved substrates, which motivated a modification of the model by adding a saturation term *β* (see details in Method "Modified Michaelis-Menten kinetics with saturation").

#### Modified Michaelis-Menten kinetics with saturation

Eq (2) is the modified Michaelis-Menten model for approximating the changing rate of one uncleaved substrate species when reacting with one protease. *β* is the saturation term representing the concentration of uncleaved substrate at which the reaction stops (when *S* = *β*, the RHS becomes 0). In subsequent discussions, we refer to V, K, n, and *β* as kinetic parameters, and [*α*] as the concentration mixing coefficient.

$$\frac{d[S]}{dt}=-[\alpha ]V\frac{{\left[S-\beta \right]}^{n}}{{\left[S-\beta \right]}^{n}+K^{n}}$$ (2)

                              [*S*] ∈ [*β*,1], [*S*]_*t = 0*_ = 1.

                              *β* ∈ [0,1]

To generalize Eq (2) for modeling the dynamics of substrate cleavage by mixtures of proteases, subscripts *i* and *j* are introduced in Eq (3), which models the changing rate of the uncleaved substrates when reacting with multiple proteases.

$$\frac{d[S_{i}]}{dt}=-\sum_{j}[\alpha_{j}]V_{ij}\frac{{\left[S_{i}-\beta ij\right]}^{n_{ij}}}{{\left[S_{i}\right]}^{n_{ij}}+{K_{ij}}^{n_{ij}}}$$ (3)

                              [*S*_*i*_], i = 1, 2, 3, …, M

                              [*α*_*j*_], j = 1, 2, 3, …, N, ∀ j: [*α*_*j*_] > = 0

[*S*_*i*_] is the amount of uncleaved substrates of the *i*^*th*^ substrate in the substrate library. [*α*_*j*_] is the concentration of *j*^*th*^ protease in the mixture. Let *M* be the number of substrates and *N* be the number of proteases. Eq (3) assumes that no synergistic or antagonistic effect is involved among various proteases within the protease mixture when cleaving substrates.

### Simulating *in silico* experiments

#### Simulating single-protease-single-substrate data

Given [*S*]_*t = 0*_ = 1, [*α*] = 1, and a set of specific kinetic parameter values (*V*, *K*, *n*, *β*), the amount of remaining uncleaved substrate [*S*]_*t = tz*_ at a specific time *t = t*_*z*_ can be calculated by numerically solving Eq (2). The kinetic parameter values are either randomly generated or estimated from real experimental data under single-protease-single-substrate setting (Method "Estimating kinetic parameters in the single-protease-single-substrate setting"). Let *Q* be the number of measurement time points, *t = t*_*z*_ (*z* = 1, 2, …, *Q*). This simulation generates a *Q*×1 data vector representing the simulated amounts of the uncleaved substrate at *Q* time points for the single-protease-single-substrate scenario.

#### Simulating data with multiple proteases

Given a library of *M* -substrates and a mixture of *N* -proteases, coefficient [*α*_*j*_] as the concentration of *j*^*th*^ protease in the mixture, and (*V*_*ij*_, *K*_*ij*_, *n*_*ij*_, *β*_*ij*_) as kinetic parameters for the reaction between the *i*^*th*^ substrate and the *j*^*th*^ protease, the amount of remaining uncleaved *i*^*th*^ substrate [*S*_*i*_]_*t = tz*_ at a specific time *t = t*_*z*_ can be calculated by numerically integrating Eq (3). The values of the kinetic parameters and mixing coefficients are either randomly generated or estimated from real experimental data (Method "Estimating mixing coefficients with multiple proteases"). This simulation generates an *M*×*Q* matrix as the simulated data.

### Estimating kinetic parameters and mixing coefficients

#### Estimating kinetic parameters in the single-protease-single-substrate setting

For each single-protease-single-substrate combination, we measure/simulate reaction products at *Q* time points after reaction starts, resulting in a data vector ***Y*** with dimension *Q*×1. In addition to simulated *in silico* experiments (Method "Simulating single-protease-single-substrate data"), ***Y*** can also be collected from real experiments under the single-protease-single-substrate setting. The problem of estimating the kinetic parameters can be formulated as the following optimization problem:

$$\underset{V,K,n,\beta }{\mathrm{minimize}}\;\sum_{z=1}^{Q}\left(y_{z}-(1-\int_{0}^{t_{z}}V\frac{[S-\beta {]}^{n}}{[S-\beta {]}^{n}+K^{n}}dt\right){)}^{2}$$ (4)

Using the “active-set” algorithm [43–45], this optimization problem leads to one set of kinetic parameters that can best fit the data ***Y*** of the specific protease-substrate combination (details see **S2 Text**). For a collection of single-protease-single-substrate settings of *M* substrates and *N* proteases, optimization is performed for each of the *M*×*N* combinations, resulting in kinetic parameter matrices (***V***, ***K***, ***n***, ***β***), each of which has a dimension of *M*×*N*.

#### Estimating mixing coefficients with multiple proteases

Once kinetic parameters for all single-protease-single-substrate combinations have been estimated, we move to estimate the mixing coefficients of proteases in a protease mixture. Let *M* be the number of substrates, *N* be the number of proteases in the mixture, and *Q* be the number of measurement times for the reaction between each substrate and the mixture. The problem of estimating the mixing coefficients can be formulated into an optimization problem as follows:

$$\underset{\alpha_{1},\ldots \alpha_{N}}{\mathrm{minimize}}\;\sum_{i=1}^{M}\sum_{z=1}^{Q}(y_{i,z}-(1-\sum_{j=1}^{N}\int_{0}^{t_{z}}\alpha_{j}V_{ij}\frac{[S_{i}-\beta_{ij}{]}^{n_{ij}}}{[S_{i}-\beta_{ij}{]}^{n_{ij}}+K^{n_{ij}}}dt){)}^{2}$$ (5)

                              [S_i_], i = 1, 2, 3, …, M

                              [*α*_*j*_], j = 1, 2, 3, …, N, ∀ j: [*α*_*j*_] > = 0

***Y*** is an *M*×*Q* data matrix either generated from *in silico* simulation (Method "Simulating data with multiple proteases") or collected from real experiment under the multi-proteases setting. This optimization problem, solved by “active-set” algorithm [43–45], generates estimations of the mixing coefficients of proteases in the mixture (details see **S2 Text**).

### Quantifying estimation accuracy via Root-Mean-Square Error (RMSE)

Once estimated mixing coefficients of a protease mixture have been obtained, the estimation accuracy is evaluated by the root-mean-square error (RMSE) metric. This metric is commonly used in machine learning to quantify accuracy for regression analysis [46]. An example of quantifying estimation error using RMSE is in **Table 2**.

**Table 2:** The first row is true *α* in P mixtures, of which each has N proteases. The second row is estimated *α*. The RMSEs for individual proteases (R_1_, …, R_N_) are calculated in the third row, and the overall RMSE will be the average of all individual RMSEs. In the simulation setting, P is the number of repetitions we applied. The repetition time is P = 200.

	Protease 1	Protease 2	…	Protease N
True *α*	$\left[\begin{array}{c} \alpha_{11}\\ \alpha_{\begin{array}{c} 12\\ \ldots \end{array}}\\ \alpha_{1P} \end{array}\right]$	$\left[\begin{array}{c} \alpha_{21}\\ \alpha_{\begin{array}{c} 22\\ \ldots \end{array}}\\ \alpha_{2P} \end{array}\right]$	…	$\left[\begin{array}{c} \alpha_{N1}\\ \alpha_{\begin{array}{c} N2\\ \ldots \end{array}}\\ \alpha_{NP} \end{array}\right]$
Estimated $\hat{\alpha }$	$\left[\begin{array}{c} {\hat{\alpha }}_{11}\\ {\hat{\alpha }}_{\begin{array}{c} 12\\ \ldots \end{array}}\\ {\hat{\alpha }}_{1P} \end{array}\right]$	$\left[\begin{array}{c} {\hat{\alpha }}_{21}\\ {\hat{\alpha }}_{\begin{array}{c} 22\\ \ldots \end{array}}\\ {\hat{\alpha }}_{2P} \end{array}\right]$	…	$\left[\begin{array}{c} {\hat{\alpha }}_{N1}\\ {\hat{\alpha }}_{\begin{array}{c} N2\\ \ldots \end{array}}\\ {\hat{\alpha }}_{NP} \end{array}\right]$
RMSE (Protease)	$R_{1}=\sqrt{\frac{\sum_{k=1}^{P}{\left({\hat{\alpha }}_{1k}-\alpha_{1k}\right)}^{2}}{P}}$	$R_{2}=\sqrt{\frac{\sum_{k=1}^{P}{\left({\hat{\alpha }}_{2k}-\alpha_{2k}\right)}^{2}}{P}}$	…	$R_{N}=\sqrt{\frac{\sum_{k=1}^{P}{\left({\hat{\alpha }}_{Nk}-\alpha_{Nk}\right)}^{2}}{P}}$
RMSE (overall)	$R_{overall}=\frac{\sum_{j=1}^{N}R_{j}}{N}$			

### Evaluating deconvolution performance and optimizing substrate selection

Given the kinetic parameter values of the reactions between a set of proteases and a set of substrates, we would like to evaluate whether we can accurately deconvolve mixtures of the proteases by measuring their cleaving activities against the substrates. We first simulate the *in silico* experimental data corresponding to the single-protease-single-substrate scenario (Eq 2) and simulate the *in silico* experimental data for protease mixtures reacting with multiple substrates (Eq 3). We then estimate the kinetic parameter values based on the simulated single-protease-single-substrate data (Eq 4). Finally, we estimate the mixing coefficients based on the estimated kinetic parameters and the simulated experimental data for protease mixtures reacting with multiple substrates (Eq 5), and evaluate the deconvolution accuracy using RMSE. In this analysis pipeline, we choose to estimate the single-protease-single-substrate kinetic parameters because the true kinetic parameter values are often unavailable in practice. Using this pipeline, we can evaluate the expected deconvolution performance for a given set of proteases using a given set of substrates and then derive optimal experimental designs for choosing the most suitable substrates for deconvolving the protease mixtures.

## Results

### Recombinant protease substrate specificity

**Fig 1:**
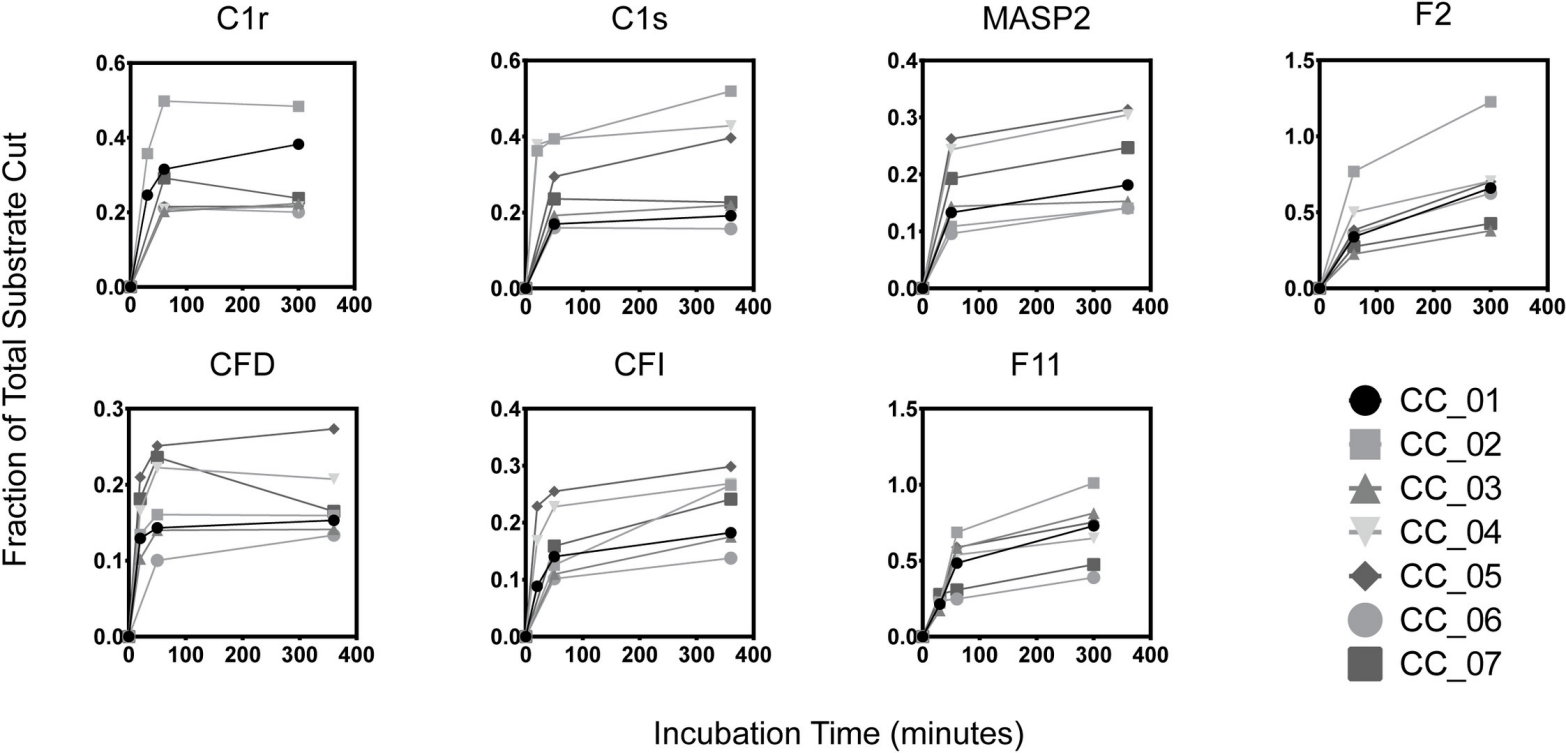
Recombinant protease cleavage assays of seven complement and coagulation cascade proteins. From left to right, top to bottom, abbreviations are: Complement proteins C1r and C1s, MASP2, Coagulation Factor IIa, Complement Factor D, Complement Factor I, and Coagulation Factor XIa. Each trace represents a different peptide substrate (CC01–07).

To obtain kinetic protease activity data we incubated 7 serum proteases from the complement and coagulation cascades with 7 protease substrates (**Fig 1**). These results showed that while each protease hydrolyzed the library of probes with different velocities, each signature was not necessarily linearly independent. Interestingly, certain proteases that showed similar activity toward the panel of substrates are involved in different physiological processes. For example, MASP2 and CFI showed similar activity signatures against this panel of substrates, although each are involved in different pathways of the complement system (e.g., MASP2 is in the lectin pathway, CFI is in the alternative pathway). In other words, this demonstrates that proteases may be related at the activity level, but may be involved in different physiological processes. Additionally, each protease showed unique early saturation levels, which we characterized with the parameter *β*.

### Validating the RMSE for evaluating protease deconvolution

To verify the efficacy of using root mean squared error (RMSE) to approximate estimation accuracy, we simulated a series of 2-protease mixtures with increasing levels of similarity between the two proteases, which represented deconvolution problems with an increasing level of difficulty. In the simulations, the number of observed time points Q was 2, which matched our experimental time points shown in **Fig 1**. More specifically, we first simulated two proteases (*p*_1_,*p*_2_) by randomly generating their kinetic parameters against multiple substrates. Since the kinetic parameters were randomly generated, these two proteases were independent of each other. We then generated a series of intermediate proteases by linearly combining the two sets of kinetic parameters: *p*_3_ = *λp*_1_+(1−*λ*)*p*_2_, *λ* = 0,0.05,0.1,0.15,…,1. After that, we (1) simulated substrate cleavage data of protein mixtures of *p*_1_ and *p*_3_ defined by varying values for *λ*, (2) performed optimizations to estimate the mixing coefficients of the mixtures, and (3) applied RMSE to evaluate the estimation accuracy. Intuitively, the estimation problem is more difficult for cases where the mixed proteases are highly correlated (*λ* close to 1). In addition, we simulated cases with varying numbers of substrates (i.e., 2–7 substrates) and, in general, the more substrates that were measured, the easier it was to deconvolve the protease mixtures. In these simulations, the RMSE is expected to be larger for more difficult cases, and smaller for relatively easier cases.

**Fig 2:**
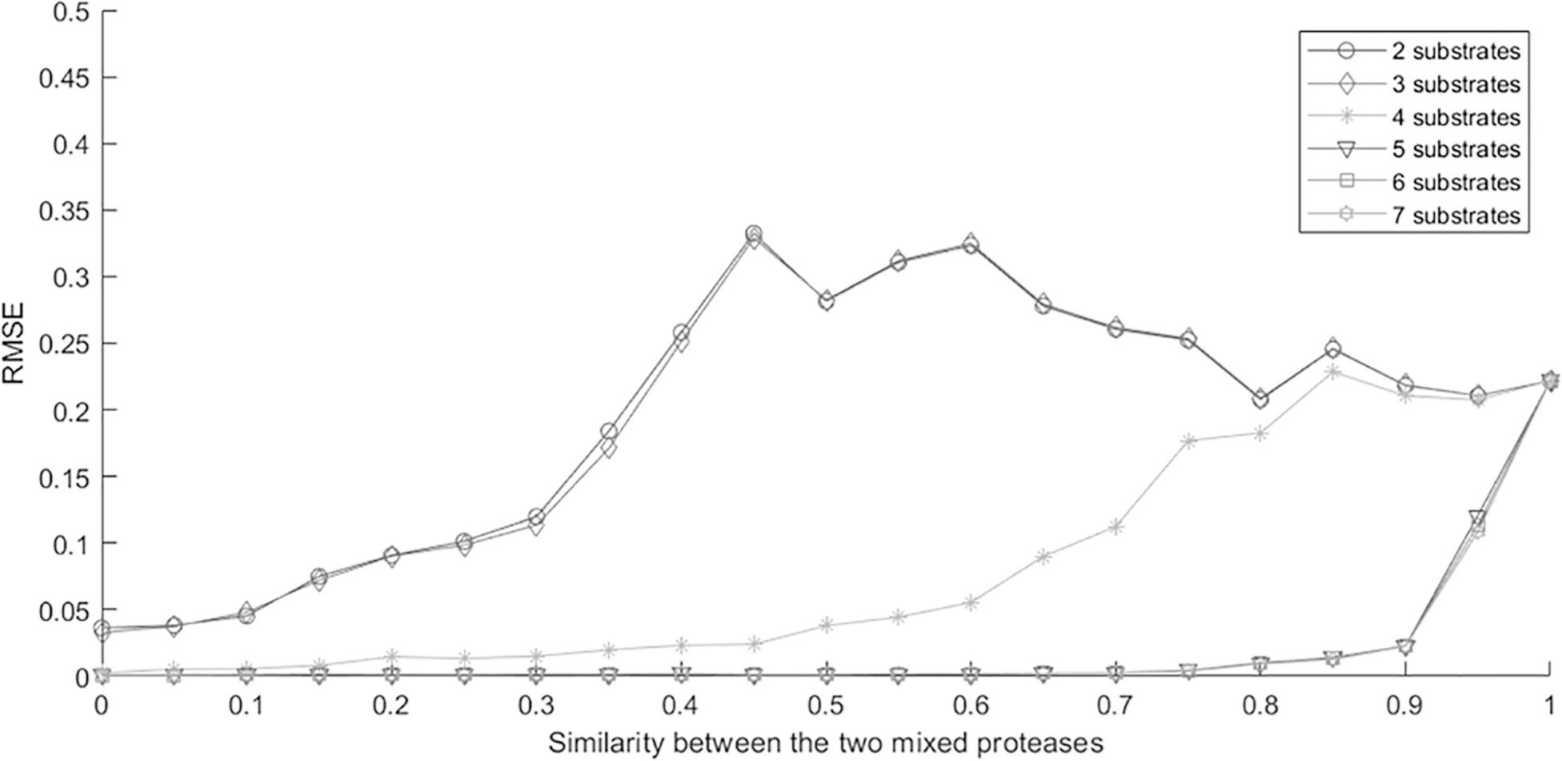
RMSE reflected the level of difficulties in deconvolution of the simulated protease mixtures. The x-axis represents the similarity between the two proteases in the mixture. A higher similarity between proteases led to a higher correlation between their cleavage dynamics, and thus higher difficulty in deconvolution. The y-axis was the RMSE of the estimated mixing coefficients. For each substrates-proteases setting, repetition P = 200 was used to calculate the corresponding reported RMSE. Each curve represented a simulation series with a different number of substrates. A smaller number of substrates corresponded to more difficult situations for deconvolution analysis.

In **Fig 2**, the horizontal axis represented the *λ* value for generating the protease *p*_3_, which meant that simulation cases from left to right had an increasing level of similarity between proteases *p*_1_ and *p*_3_, and thus had an increasing level of difficulty for deconvolving protease mixtures of the two proteases. Each curve represented a different series of simulations with a particular number of substrates. In **Fig 2**, the simulation series with a larger number of substrates led to smaller RMSEs. Note that the 2- and 3-substrates curves largely overlapped, and the 5-, 6-, and 7-substrates curves also largely overlapped. In each simulated series with a specific number of substrates, the RMSE increased in general with respect to the horizontal axis that represented an increasing level of difficulty. In the 2- and 3-substrates curves, the changes of RMSE were not monotonic. This was mainly because, with a limited number of substrates in the simulation, *λ* around 0.5 already represented quite difficult situations that led to very large RMSE with high variance. For the subsequent larger *λ* values representing even more difficult situations, the slight decrease of the subsequent RMSEs was due to the high variance when the RMSE was large. Overall, the observed RMSEs showed expected trends with respect to the level of difficulty of the simulated cases, validating that the RMSE is a useful evaluation metric.

### Optimizing choices of substrates

**Fig 3:**
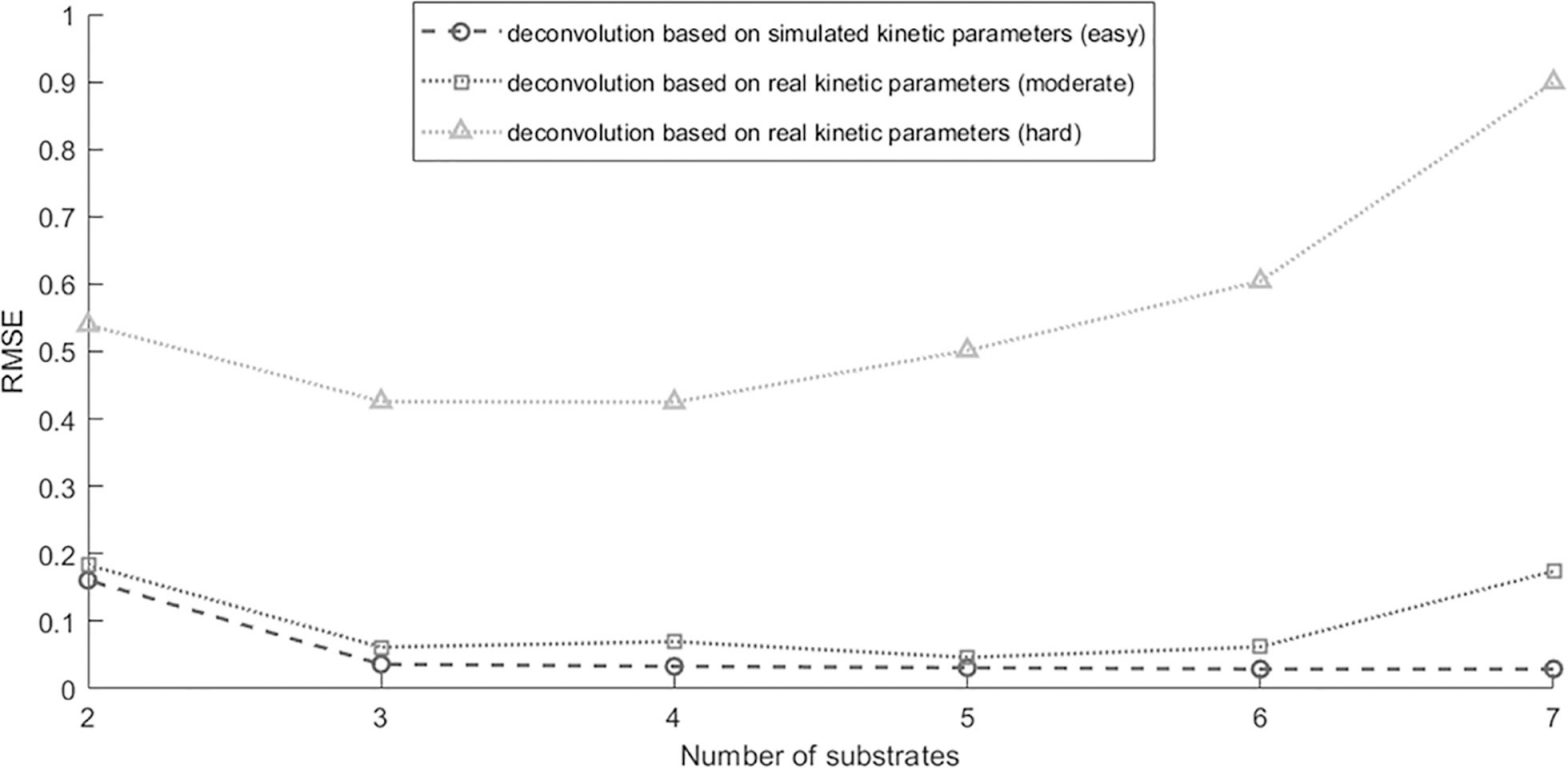
Influence of substrate library’s size on the deconvolution accuracy. The x-axis represented the number of substrates applied for deconvolution and the y-axis represented the resulting RMSE. The dashed curve with circles represented RMSE for deconvolving mixtures of three simulated proteases that are independent, and showed increased RMSE as the number of substrates decreased from 7 down to 2. The dotted curves with squares and with triangles represented RMSE for deconvolving mixtures of three real proteases that are slightly correlated and highly correlated accordingly, where the RMSEs were relatively high even when the number of substrates was 7. Repetition time P = 200 was applied for each substrates-proteases setting. Discussion regarding the decrease of RMSE when reducing the number of substrates were discussed in **Supplementary Figure B—G**.

To demonstrate the feasibility of optimizing choices of substrates, we considered 3 proteases and 7 substrates, with their single-protease-single-substrate kinetic parameters randomly generated. We first evaluated the accuracy for deconvolving mixtures of the 3 proteases using all 7 substrates, which resulted in low RMSE as shown by the right-most point on the dashed-circle line in **Fig 3**. We then removed one substrate and evaluated the RMSE for deconvolution with 6 substrates. All 7 possibilities were evaluated, and the best RMSE was reported as the second right-most point on the dashed line, which was virtually the same as the 7-substrate scenario. We iterated this analysis, removing one substrate that had the least impact on RMSE in each iteration, until only 2 substrates remained. As shown in **Fig 3**, the RMSE remained low until the number of substrates reduced from 3 to 2. This was because the single-protease-single-substrate kinetic parameters were randomly generated, which represented 3 proteases that had independent substrate cleavage activities. In other words, at least three substrates were needed to estimate the activity of 3 independent proteases.

We performed two sets of similar analyses using 3 of the 7 proteases and the 7 substrates in our real experimental data in section "Recombinant Protease Substrate Specificity". One set of analyses was based on proteases MASP2, C1r, and F2, which were from 3 different proteases families, and the other set of analyses was based on proteases MASP2, CFI, and CFD, which were highly correlated in terms of their substrate cleavage dynamics. The single-protease-single-substrate kinetic parameters were estimated from real experimental data. All subsequent analyses were the same as the above where kinetic parameters were randomly generated. As shown by the dotted-triangle curve in **Fig 3**, deconvolving the three highly correlated proteases was quite difficult with large the RMSE regardless of how many substrates were used. The dotted-square curve in **Fig 3** was similar to the analysis where kinetic parameters were randomly generated, indicating that the three proteases from different protease families had relative independent cleavage dynamics against the substrates. Interestingly, the performance actually improved in both dotted curves when the number of substrates reduced from 7 to 5 (or 4). This was because the first few substrates being removed had extremely similar cleavage dynamics against all the proteases (details in **Figs B-G in S3 Text**). Those substrates were not only uninformative but also sources of confusion for the deconvolution analysis. Therefore, effective deconvolution of protease mixtures required a decent number of substrates with uncorrelated cleavage dynamics against the proteases. However, correlated substrate cleavage dynamics is ubiquitous, especially among proteases in the same physiological family. When deconvolving mixtures containing highly correlated proteases, even a large number of substrates may not lead to satisfactory deconvolution performances. This motivated us to investigate a less ambitious goal of deconvolving protease families, instead of deconvolving individual proteases.

### Deconvolving protease families

As mentioned above, deconvolving protease mixtures can be challenging and may require an impractically large number of substrates when proteases with highly correlated substrate cleavage dynamics exist in the mixture. Therefore, we proposed to verify the efficacy of clustering highly correlated proteases into families, and deconvolving the activity signatures by estimating the mixing concentration of the protease families, rather than individual proteases.

### Deconvolving simulated protease families

We first simulated a scenario with 9 proteases and 7 substrates, in which the 9 proteases formed three families. Each family contained 3 highly correlated proteases, but the families were independent of each other. To generate the 3 highly correlated proteases in one family *in silico*, we took a similar strategy as described in Section "Validating the RMSE for evaluating protease deconvolution". For each protease family, we randomly generated the kinetic parameters of 3 proteases (*p*_1_, *p*_2_, and *p*_3_) cleaving 7 substrates, and then generated 2 proteases *p*_4_ and *p*_5_ that correlated *p*_1_ using the following combinations *p*_4_ = *λp*_1_+(1−*λ*)*p*_2_, and *p*_5_ = *λp*_1_+(1−*λ*)*p*_3_. Proteases *p*_1_, *p*_4_ and *p*_5_ form the family. Here, *λ* was either 0.9 or 0.6, representing a proteases family containing highly correlated proteases or moderately correlated proteases. We repeated the above three times to generate the kinetic parameters for the three families of proteases.

**Fig 4:**
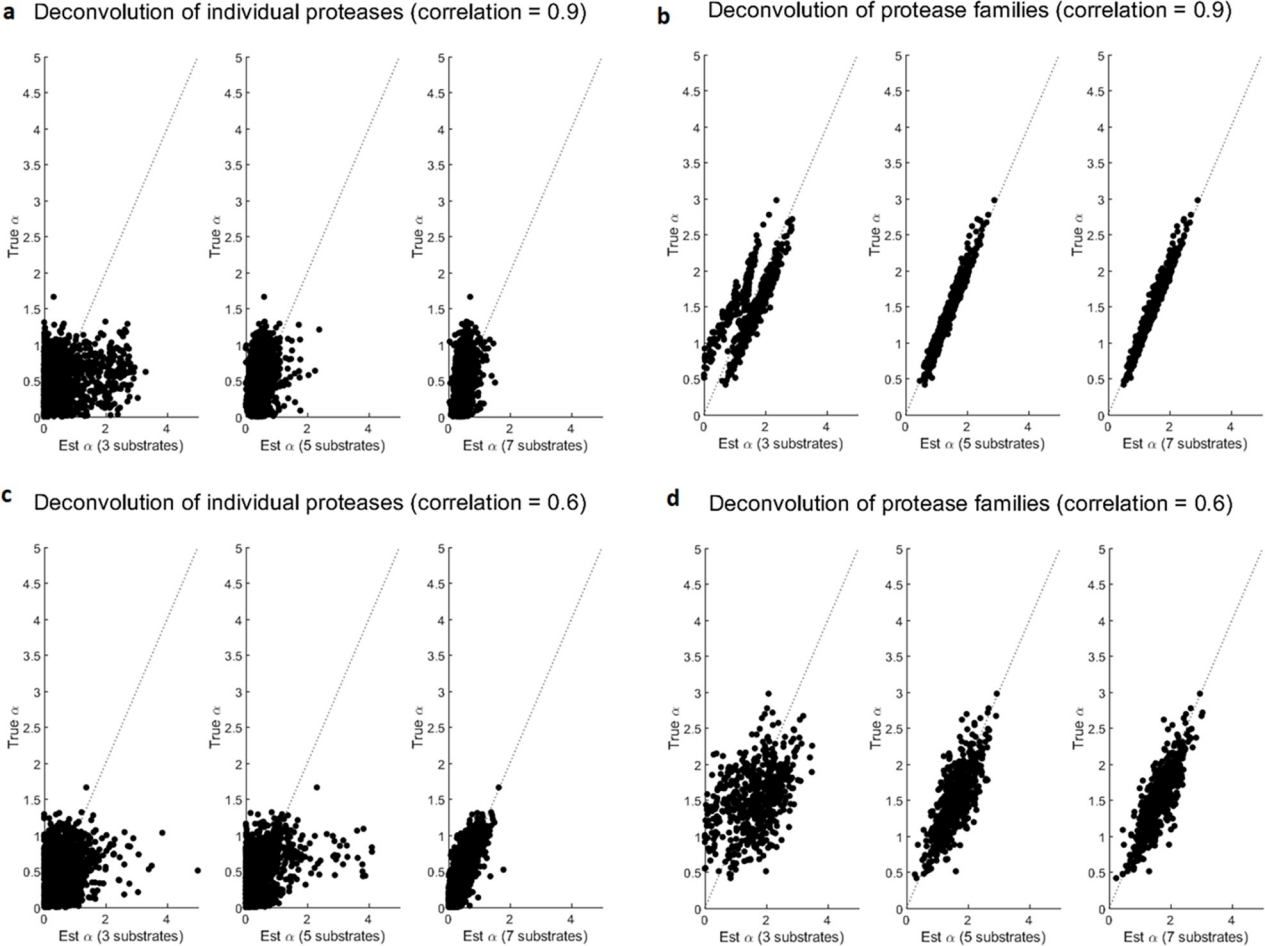
Comparison between deconvolution of individual proteases and deconvolution of protease families. The x axis represented the estimated mixing coefficients. The y axis represented the true simulated mixing coefficients. Consider mixtures of 9 proteases from 3 independent families that contain highly correlated proteases within each family. **(a)** When deconvolving the 9 individual proteases, the estimated mixing coefficients for the individual proteases showed poor agreements with their true simulated values, regardless of whether 3, 5, or 7 substrates were used. **(b)** When deconvolving the 3 protease families, the estimated mixing coefficients for the protease families showed high agreement with the sum of the family members’ true mixing coefficients. **(c-d)** Simulation of protease families that contain moderately correlated proteases showed similar results, where deconvolution of individual proteases was difficult but deconvolution of protease families was accurate.

After generating the single-protease-single-substrate kinetic parameters with family structures, we simulated data for the single-protease-single-substrate setting and the multi-proteases-multi-substrates setting. We then evaluated the performance for deconvolving the 9 individual proteases using 3, 5, or 7 substrates. **Fig 4A and 4C** showed scatter plots of the true simulated mixing coefficients versus the estimated mixing coefficients, where the estimation performance was poor. The only exception was the case in the third plot of **Fig 4C**, where the protease was moderately correlated (*λ* = 0.6) and the number of substrates was 7. This was the least challenging case simulated here for deconvolving individual proteases, where the estimated mixing coefficients roughly tracked the true mixing coefficients. Overall, with the presence of correlated proteases, although the protease families were independent, it was difficult to deconvolve the mixing coefficients of the individual proteases.

Using the same simulated data as above, we evaluated the possibility for deconvolving protease families. In order to perform deconvolution at the protease family level, we used Eq (4) to estimate one set of kinetic parameters for each family, by treating the simulated single-protease-single-substrate data for protease members in the same family as replicates of a "representative" protease for the family. After estimating the kinetic parameters for the three protease families, we then optimized Eq (5) to estimate the mixing coefficient of the protease families. Ideally, the estimated mixing coefficient for one protease family should approximate the sum of the true mixing coefficients of members in the family, which was indeed what we observed in the results shown in **Fig 4B and 4D**. Meanwhile, the performance difference between **Fig 4B and 4D** indicated that successful deconvolution of protease families required members within each family to be decently correlated. This analysis demonstrated the feasibility of accurately deconvolving protease families, while the deconvolution of individual proteases was difficult.

### Deconvolving protease families derived from real data

**Fig 5:**
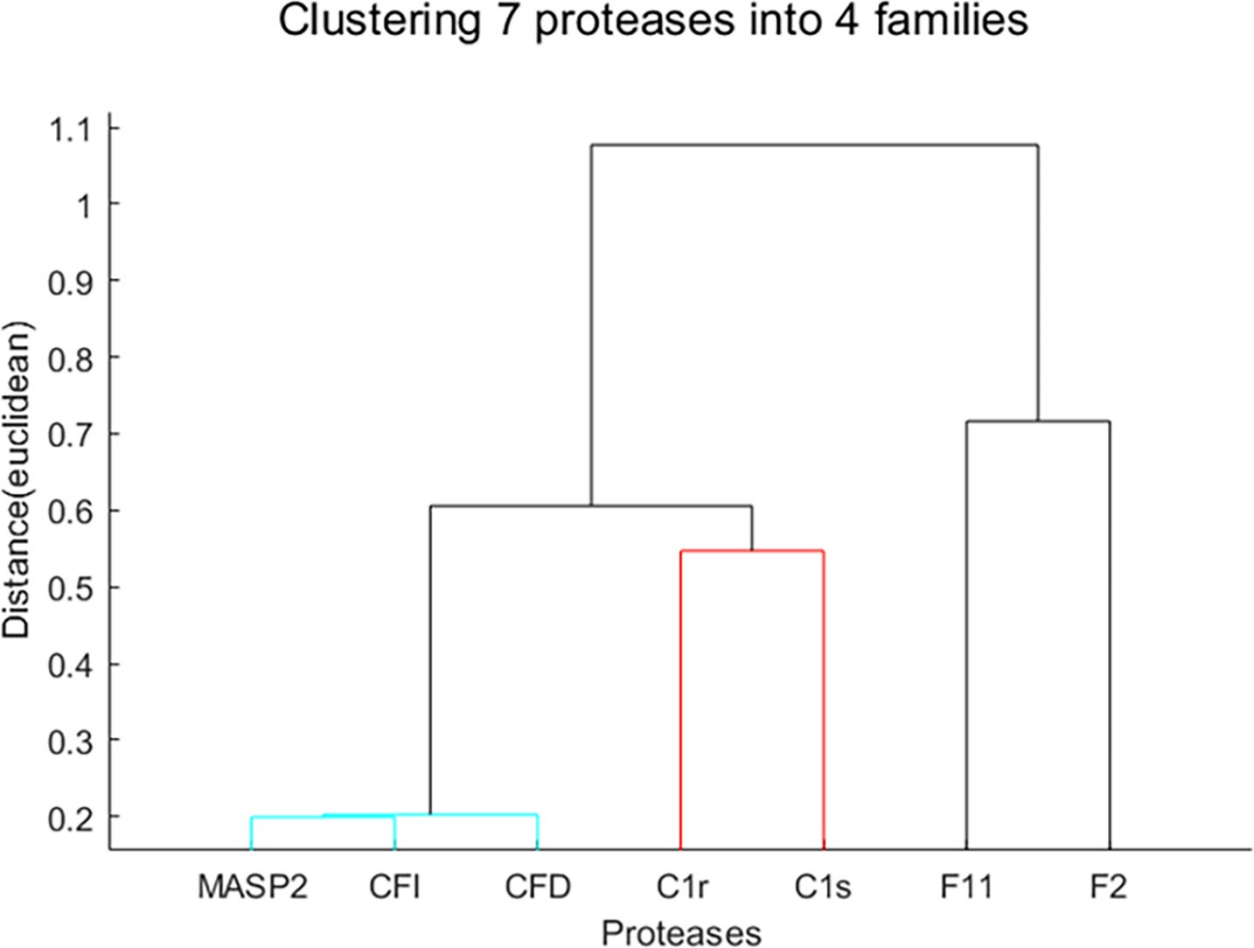
Hierarchical clustering of individual proteases led to 4 families. MASP2+CFI+CFD formed the most strongly correlated family, C1r + C1s formed the moderately correlated family, and F11 and F2 each served as its own family.

To further validate the idea of deconvolving protease families, we used the real single-protease-single-substrate data in Section "Recombinant Protease Substrate Specificity". We represented each protease by a vector containing the collection of all data points for this protease in the single-protease-single-substrate assays in **Fig 1**. We then clustered proteases by agglomerative hierarchical clustering using single linkage and Euclidean Distance. To group the proteases into families, we chose a cut-off value for distance to be 0.6. This allowed us to cluster proteases with correlated hydrolytic activities into the same family. After the clustering analysis, we grouped the 7 individual proteases into 4 computationally-derived families: (1) Complement protein MASP2 (lectin pathway) and Complement Factor I and D (alternative pathway), (2) Complement proteins C1r and C1s (classical pathway), (3) Coagulation factor IIa, and (4) Coagulation factor XIa. (**Fig 5**). Our results showed that the computationally-derived clusters reflect the physiological pathways they are involved in (i.e., group 1 and 2 are complement pathways and group 3 and 4 are coagulation pathways). This is also reflected at the sequence level, where clustered proteases C1r and C1s share 38.5% sequence identity while C1s shares only 27.9% and 31.2% identity with CFD and F11, respectively [47].

**Fig 6:**
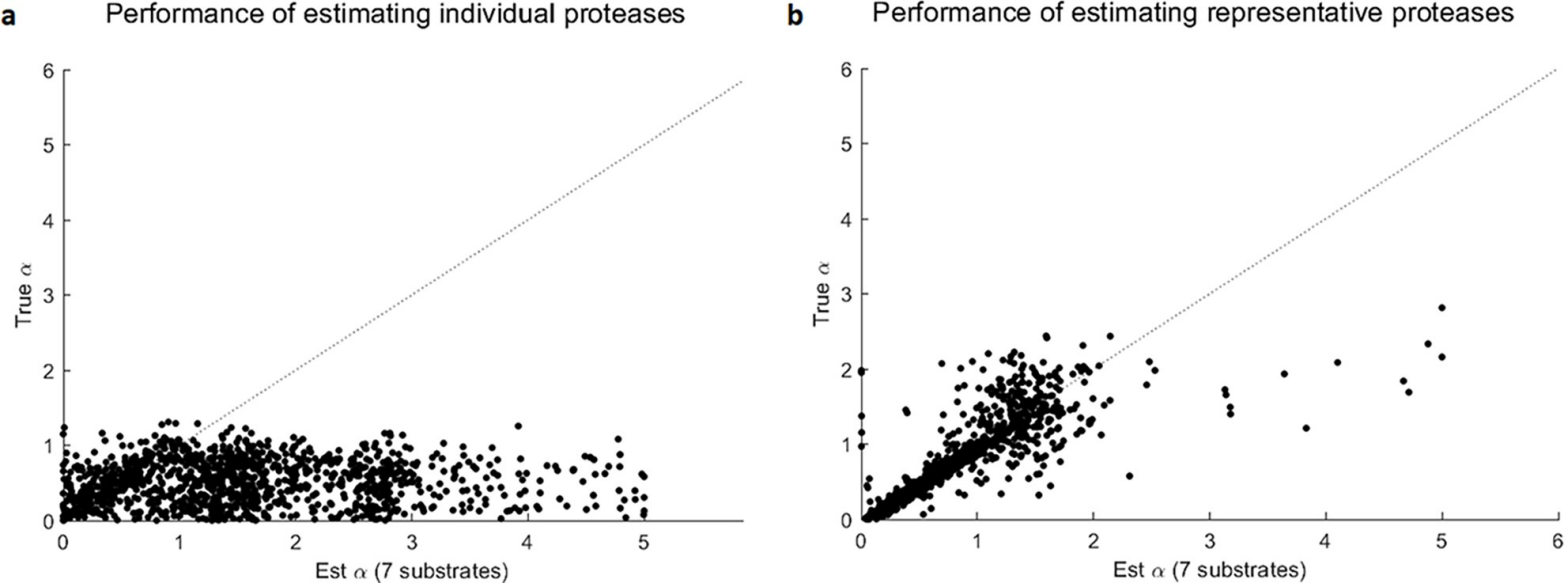
Performances of estimating representative families and individual proteases using the same 7 proteases. **(a)** Deconvolution of 7 individual proteases was no better than a random guess since the scatter dots spread out widely on the horizontal direction. **(b)** After clustering 7 individual proteases into 4 protease families as shown in Fig 5, deconvolution accuracy increased, with mixing coefficients of protease families being close to the sum of individual mixing coefficients within according protease families.

After that, we performed the same analysis as above, including simulation of the multi-protease-multi-substrate data, estimation of the kinetic parameters for the protease families, and estimation of the mixing coefficients for the protease families. We also performed deconvolution for the individual proteases. As shown in **Fig 6**, the deconvolution of the individual proteases performed poorly, while the estimated mixing coefficients of the protease families decently tracked the simulated true mixing coefficients. The deconvolution accuracy of these protease families was lower than the above analysis of randomly generated kinetic parameters. This was because the randomly generated kinetic parameters led to simulated protease families that were independent of each other, whereas the protease families derived from real data were not as independent. Since human proteases are known to organize in a nested hierarchy of protease families and subfamilies, the correlation among them makes deconvolution of individual protease cumbersome and impractical. This analysis provides a practical alternative for sensing and characterizing protease activities at the level of protease families.

## Discussion

While significant advances in measuring large amounts of biological information have been made by using genome-wide sequencing techniques, the lack of correlation between expression and activity is a major limitation [13]. Further, the primary drivers of physiological processes in health and disease are enzymes (e.g., proteases, kinases), meaning valuable information is stored in the real-time activity of these proteins. One of the major challenges in scaling up to multiplexed libraries for protease activity analysis is substrate design, due to the difficulty of screening for specific substrates. Due to the promiscuity of proteases, the goal of designing substrates with both high sensitivity and high specificity for all human proteases is experimentally challenging. To date, the major advancements in this substrate design include multiplex substrate profiling by mass spectrometry (MSP-MS) [48–50] and randomized substrate screens with phage display [51], as well as computational approaches for receptor-ligand prediction [52, 53]. Here, we present a framework for evaluating the activity contributions of individual proteases within a complex mixture with minimal substrate design. However, the MSP-MS and phage display methods provide the ability to develop peptide substrates for proteases *ad hoc*, which we envision could be combined with our algorithm to enable more strategic control over the foundational protease signatures. Further, these methods provide the ability to identify the dominant proteases in a biological sample, which could also be used to pre-emptively narrow our list of target proteases.

Fundamentally, however, human proteases are organized in a hierarchy of protease families that consist of proteases with highly correlated activities against many substrates. Deconvolution of highly correlated proteases would require an impractically large number of substrates. Therefore, we propose to cluster highly correlated proteases into families, and estimate the relative activity contributions of these families. We demonstrated the feasibility of accurately deconvolving protease families when the deconvolution of individual proteases was difficult. We envision that this may allow for the rapid characterization and investigation of physiologically relevant protease families, which can effectively screen the entire protease landscape before homing in on specific protease targets.

## Supporting information

S1 Text Supporting text and figures related to the screening of protease substrates. (DOCX)

S2 Text Supporting text and figures related to parameter optimization. (DOCX)

S3 Text Supporting text and figures related to deconvolution performance. (DOCX)

S1 File Example MATLAB code. (ZIP)
