# Supplementary material for: Deconvolving multiplexed protease signatures with substrate reduction and activity clustering
Source: PLoS Comput Biol. 2019 Sep 3;15(9):e1006909. doi: 10.1371/journal.pcbi.1006909 (PMC6743790; doi:10.1371/journal.pcbi.1006909)
Supplement: S1 Text — (DOCX) [file pcbi.1006909.s001.docx]

**Supplementary Text S1**

**S1.1 Screening for Protease Substrates**

As mentioned in section "Recombinant Protease Activity Assay", we originally screened 20 candidate peptide substrates for proteases that cleave after an arginine residue. These substrates were found in the literature, either in the context of phage display studies or studies of the proteases' physiological substrate (*37-40*). For example, the sequences LQRIYKC and HRGRTLEIC were identified with phage display screens, whereas RALERGLQDC, GLQRALEIC, and SSTGRNGFKC were identified from the physiological substrate, complement protease C4. Additionally, the sequence KSVARTLLVKC was identified from the physiological C1 inhibitor and QRQRIIGGC was identified by analyzing the mechanism of C1r autocatalysis. Sequences LPSRSSKIC, HRGRTLEIC, and STGRNGFKC were identified from C3 and C4b precursor target substrates and QQKRKIVLC is a physiological substrate present on Factor B (*37-40*). The proteases tested include 5 of the complement proteases used in later activity assays. The sequences were modified on the N-terminus with the fluorophore 5-FAM, and synthesized with a c-terminal cysteine to be conjugated to amine-modified iron oxide nanoparticles by SIA (i.e., succinimidyl iodoacetate) reaction for efficient fluorophore self-quenching. Upon proteolytic hydrolysis, the fluorophore was released and an increase in signal was observed (i.e., fold-change in relative fluorescence units, FC RFU). All samples were incubated with a peptide concentration of 2 μ M and incubated at 37°C for 60 minutes. From these sequences, we chose 7 substrate sequences that in total would be hydrolyzed well by all candidate proteases (**Fig A**).

**
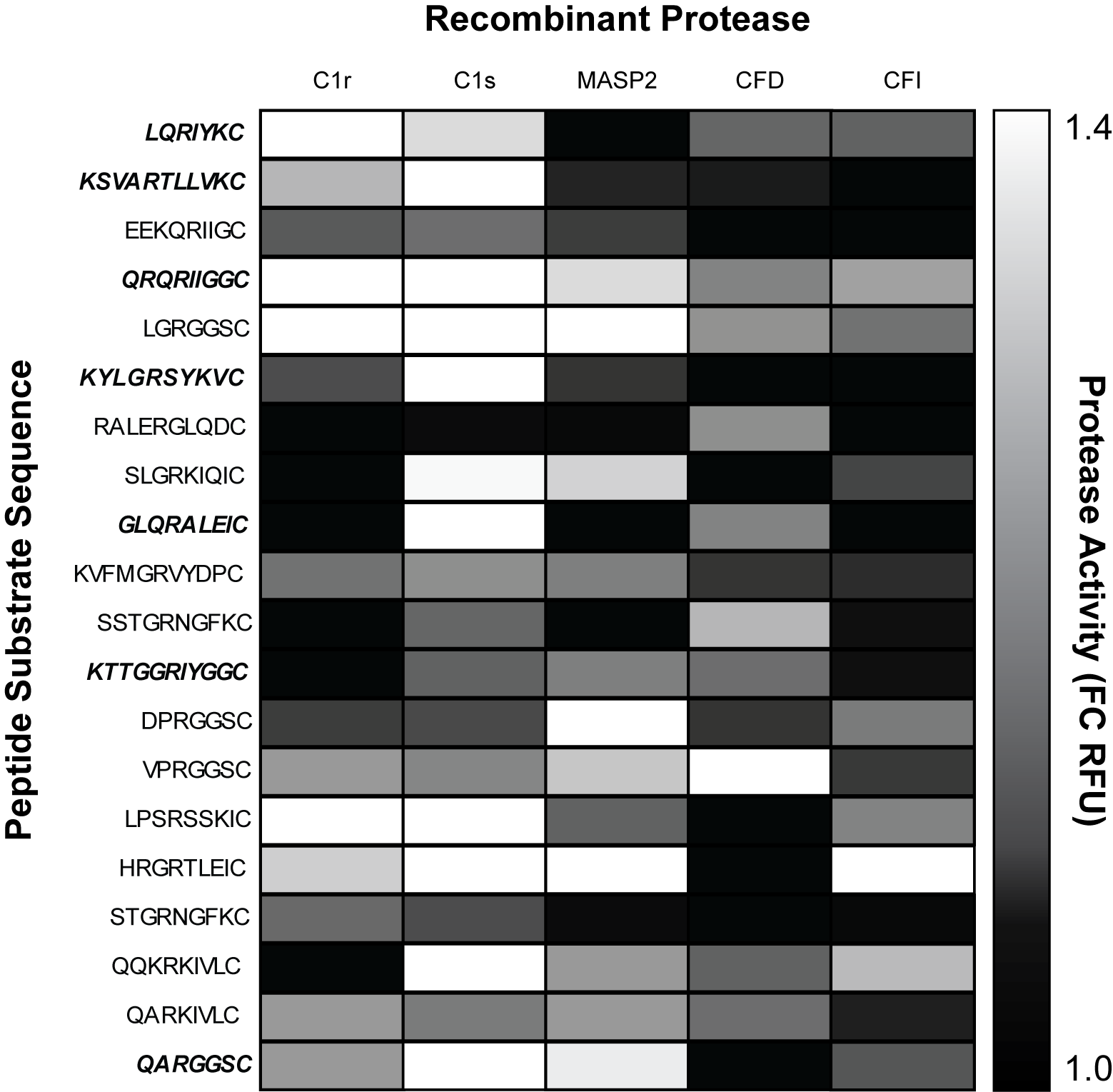
**

**Figure A.** Heatmap of protease-substrate activity assay for 20 candidate peptide sequences and 5 recombinant complement proteases. Proteases in bold/italic were the sequences chosen to be used for the main activity studies in the manuscript.
