# Supplementary material for: Deconvolving multiplexed protease signatures with substrate reduction and activity clustering
Source: PLoS Comput Biol. 2019 Sep 3;15(9):e1006909. doi: 10.1371/journal.pcbi.1006909 (PMC6743790; doi:10.1371/journal.pcbi.1006909)
Supplement: S2 Text — (DOCX) [file pcbi.1006909.s002.docx]

**Supplementary Text S2**

**S2.1 Numerically optimizing parameter choices**

As mentioned in **Method 2.4**, the problem of estimating the kinetic parameters and mixing coefficients were formulated as optimization problems (4) and (5) (see **Method 2.4**). We numerically solved these optimization problems using the nonlinear programming solver *fmincon* (*43-45*) in Matlab. Two of the parameters required by *fmincon* are worth mentioning here. The first one was the optimization algorithm applied (where we chose “active-set” algorithm), and the second one was the set of upper/lower bounds for the optimization variables (i.e. kinetic parameters, saturation levels, and mixing coefficients here).

**S2.1.1** Choice of optimization algorithm

During our initial explorations in this study, we evaluated multiple well-established optimization algorithms, including active-set, interior-point and levenberg-marquardt. Although these algorithms use different optimization strategies (i.e., how to handle the parameter update directions, constraints and stopping criteria), their performance were highly similar. They all performed poorly in challenging cases (e.g. **Figure 4a, 4c, 5a**), and achieved satisfactory performance in relatively easy cases (e.g. **Figure 4b, 4d, 5b**). We chose to present the results based on the “active-set” algorithm because it converged slightly faster than the other two algorithms in our optimization problems.

**S2.1.2** Upper/lower bounds for the optimization variables

When estimating the kinetic parameters *V, K, n, β* here from the single-protease-single-substrate data, the kinetic variables *V, K,* were bounded in the range [e^-10^, e^10^], the kinetic order *n* was bounded in [1, e^10^], and the saturation level *β* was bounded in the range [e^-10^, 1]. These bounds were chosen because of the variables physical meanings: all kinetic parameters should be non-negative, and the saturation level is between 0 and 1. When estimating the mixing coefficients, the values for the mixing coefficients *α* were bounded in the range of [e^-10^, e^5^]. This was a generous range that allowed the algorithm to estimate mixtures that contained high concentrations of proteases. We used these upper/lower bounds in all the simulations and analyses presented in this study.
