# Supplementary material for: Deconvolving multiplexed protease signatures with substrate reduction and activity clustering
Source: PLoS Comput Biol. 2019 Sep 3;15(9):e1006909. doi: 10.1371/journal.pcbi.1006909 (PMC6743790; doi:10.1371/journal.pcbi.1006909)
Supplement: S3 Text — (DOCX) [file pcbi.1006909.s003.docx]

**Supplementary TextS3**

**Figure B.** Substrate cleavage dynamics generated by simulated kinetics parameters for a 7-substrates-3-proteases setting. The x-axis represented the reaction time, and the y-axis represented the amount of reaction product (cleaved substrates). The amounts of cleaved substrates at t = [0 30 60 300] were collected for subsequent analysis. The 3 generated proteases showed independence in terms of their substrate cleavage dynamics.

**Figure C.** Deconvolution performance of the 7-substrates-3-proteases setting using simulated kinetic parameters. The x-axis represented the estimated mixing coefficients. The y-axis represented the true simulated mixing coefficients. The deconvolution performance stayed quite decent along the way of reducing the number of substrates from 7 to 3, and showed a decrease in accuracy when reducing from 3 substrates to 2 substrates. To deconvolute 3 independent proteases, the sufficient number of proteases was 3. The deconvolution difficulty level of this independent-proteases setting was regarded as “easy” in Figure 3.

**Figure D.** Substrate cleavage dynamics generated by estimated kinetics parameters from a 7-substrates-3-proteases setting in the real experimental data. The involved proteases (from different families) were C1r, F2, and MASP2. The x-axis represented the reaction time, and the y-axis represented the amount of reaction product (cleaved substrates). The amounts of cleaved substrates at t = [0 30 60 300] were collected for subsequent analysis. The three proteases showed a moderate level of correlation in terms of their substrate cleavage dynamics, especially for substrates CC_03 and CC_07.


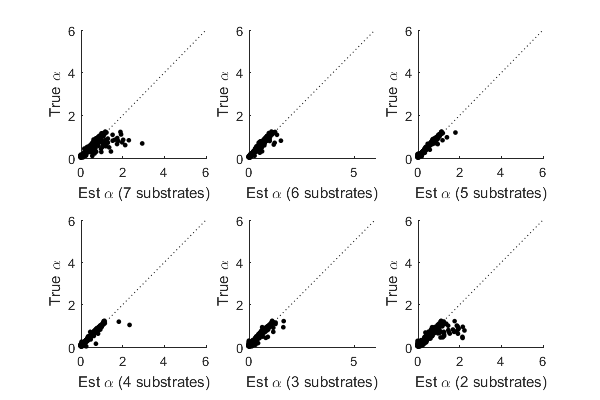


**Figure E.** Deconvolution performance of the 7-substrates-3-proteases setting using kinetic parameters estimated from real experimental data. The involved proteases were C1r, F2, and MASP2. The x-axis represented the estimated mixing coefficients. The y-axis represented the true simulated mixing coefficients. The deconvolution accuracy increased when reducing from 7 substrates to 6 and to 5 substrates, and decreased when reducing from 4 substrates to 3 and 2 to substrates. This counterintuitive observation could be explained by the high correlation among three proteases when cleaving substrates CC_03 and CC_07 in Figure S3. Removing CC_03 and CC_07, which could be regarded as “noise”, decreased the overall level of correlation and thus increased the deconvolution performance (from 7 substrates to 6 and to 5 substrates). With the correlation among proteases being reduced, further removing the number of substrates from 4 to 3 and to 2 decreased the deconvolution accuracy due to loss of information. The deconvolution difficulty level of this moderately-correlated setting was regarded as “moderate” in Figure 3.

**Figure F.** Substrate cleavage dynamics generated by estimated kinetics parameters from a 7-substrates-3-proteases setting in the real experimental data. The involved proteases (from the same family) were MASP2, CFD, and CFI. The x-axis represented the reaction time, and the y-axis represented the amount of reaction product (cleaved substrates). The amounts of cleaved substrates at t = [0 30 60 300] were collected for subsequent analysis. The three proteases showed a high level of correlation in terms of their substrate cleavage dynamics, for almost all substrates.


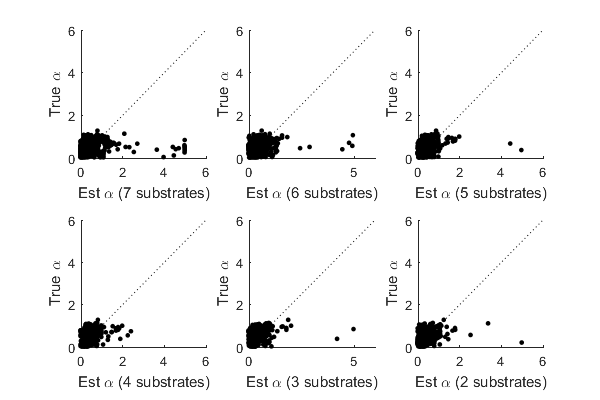


**Figure G.** Deconvolution performance of the 7-substrates-3-proteases setting using kinetic parameters estimated from real experimental data. The involved proteases (from the same family) were MASP2, CFD, and CFI. The x-axis represented the estimated mixing coefficients. The y-axis represented the true simulated mixing coefficients. The deconvolution stayed poor regardless of the number of substrates being applied. Similar to Figure S4, the slight increase in deconvolution accuracy when reducing from 7 substrates down to 4 substrates was observed again. The deconvolution difficulty level of this highly-correlated setting was regarded as “hard” in Figure 3.
